# Prenatal diagnosis of 18p deletion and 8p trisomy syndrome: literature review and report of a novel case

**DOI:** 10.1186/s12905-024-03081-4

**Published:** 2024-04-15

**Authors:** Maria Papamichail, Anna Eleftheriades, Emmanouil Manolakos, Adamantia Papamichail, Panagiotis Christopoulos, Gwendolin Manegold-Brauer, Makarios Eleftheriades

**Affiliations:** 1 Postgraduate Programme “Maternal Fetal Medicine” Medical School, National & Kapodistrian University of Athens, Athens, Greece; 2 Department of Obstetrics and Gynaecology, Women’ Hospital, University Hospital of Basel, University of Basel, Basel, Switzerland; 3 Clinical Laboratory Genetics, Access To Genome (ATG), Athens-Thessaloniki-Greece, Athens, Greece; 4 Department of Pediatriacs, General Hospital of Larnaka, Larnaka, Cyprus; 5 2nd Department of Obstetrics and Gynecology, Medical School, Aretaieio University Hospital, National & Kapodistrian University of Athens, Athens, Greece; 6 Department of Gynaecological Ultrasound and Prenatal Diagnostics, Women’ Hospital, University Hospital of Basel, University of Basel, Basel, Switzerland

**Keywords:** 18p deletion syndrome, Trisomy 8p syndrome, Prenatal diagnosis

## Abstract

18p deletion syndrome constitutes one of the most frequent autosomal terminal deletion syndromes, affecting one in 50,000 live births. The syndrome has un-specific clinical features which vary significantly between patients and may overlap with other genetic conditions. Its prenatal description is extremely rare as the fetal phenotype is often not present during pregnancy. Trisomy 8p Syndrome is characterized by heterogenous phenotype, with the most frequent components to be cardiac malformation, developmental and intellectual delay. Its prenatal diagnosis is very rare due to the unspecific sonographic features of the affected fetuses. We present a very rare case of a fetus with multiple anomalies diagnosed during the second trimester whose genomic analysis revealed a 18p Deletion and 8p trisomy Syndrome. This is the first case where this combination of DNA mutations has been described prenatally and the second case in general. The presentation of this case, as well as the detailed review of all described cases, aim to expand the existing knowledge regarding this rare condition facilitating its diagnosis in the future.

## Introduction - background

18p deletion syndrome (18p – Syndrome, [OMIM] #146,390) is one of the most frequent autosomal terminal deletion syndromes [1]. It is also the first reported partial monosomy compatible to life [2]. It was first described by de Grouchy et al. in 1963 and since then, more than 159 cases have been reported. [3, 4]. It affects one in 50,000 live births, with a female to male ratio 3:2. The syndrome has un-specific clinical features which vary significantly between patients and also they overlap with other genetic conditions [4]. Prenatal description of the syndrome is extremely rare and its diagnosis is really challenging, as in the most of the cases, the fetal phenotype of the syndrome is not present during pregnancy [5].

Trisomy 8p Syndrome was first described in 1973 by Lubs and Lubs [6, 7] and it’s prevalence is reported as 1 in 58,000 live births. This syndrome is also characterized by heterogeneity and variable phenotype, with the most frequent components to be cardiac malformation, developmental and intellectual delay [8]. Interestingly, the prenatal diagnosis of trisomy 8p is very rare due, to the unspecific sonographic features of the affected fetuses.

Herein, we present a case of a woman whose first pregnancy was terminated due to holoprosencephaly (HPE) and the second ended with a spontaneous miscarriage during the first trimester. In the last pregnancy, the fetus had multiple anomalies diagnosed during the second trimester and genomic analysis revealed a 18p Deletion and 8p trisomy Syndrome due to a paternal balanced reciprocal translocation between the short arm of chromosome 8 and the short arm of chromosome 18. This is the first time where chromosomal rearrangement has been described prenatally and the second case in general. Additionally, the presentation of the affected fetus’ phenotype will expand the knowledge of the prenatal presentation and therefore the diagnosis of this syndrome will be facilated in the future.

## Case Presentation

A 32-year-old pregnant woman (gravid 4, parity 1) presented for the routine first trimester scan at 12^+ 2^ weeks of gestation. The mother was not a smoker, her BMI (body mass index) was within normal ranges (BMI: 23,9 kg/m^2^) and she had not history of hypertensive disorders of pregnancy, autoimmune disorders or gestational diabetes mellitus. However, concerning her obstetric history, there was a pregnancy which was terminated due to holoprosengephaly, diagnosed in the first trimester scan. The parents denied an invasive diagnostic test with CVS (chorionic villus sampling) and fetal karyotype at that time. The second pregnancy ended with a spontaneous miscarriage during the first trimester. The parents did not proceed to an autopsy of neither of the fetuses. The third pregnancy was uncomplicated and ended with the delivery of a healthy boy, weighting 3860 g in 39^+ 1^ weeks of gestation.

**Fig. 1:**
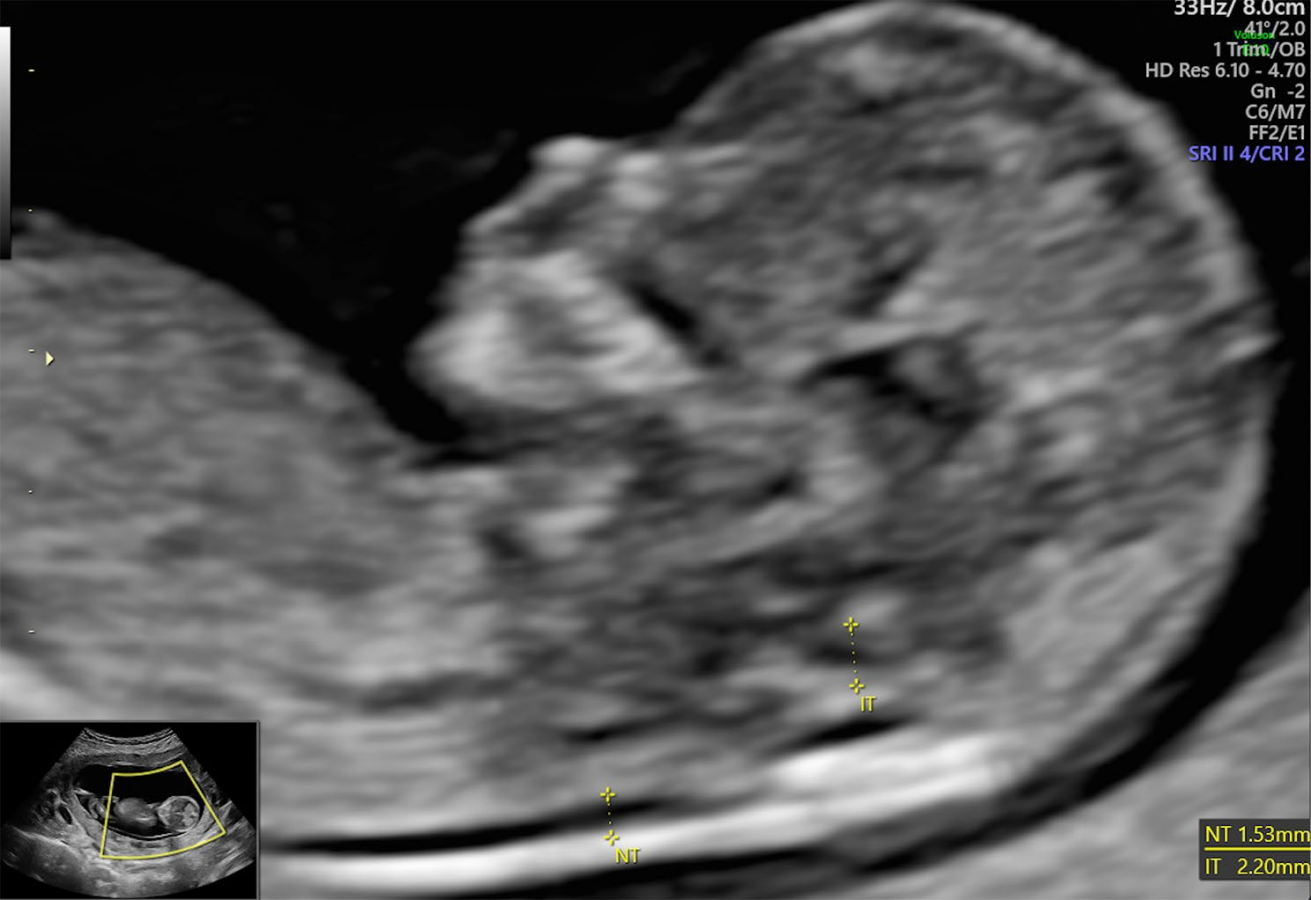
Depiction of normal NT (nuchal translucency) during first trimester

In the fourth pregnancy, the routine scan in first trimester revealed a CRL (Crown-rump Length) in accordance with the last menstrual period, while biparietal diameter, abdominal circumference and femoral length were within normal ranges for the gestational age. Assessment of brain structure, abdominal wall and renal system showed no signs of malformations. There was normal heart rate, nuchal translucency of 1,53 mm (Fig. 1), visible nasal bone and no tricuspid regurgitation. The only remarkable measure was ductus venosus pulsality index (DV PI) which was 1,26 and equal to the 95th centile. Regarding maternal biochemistry, PAPP-A (Placenta Associated Plasma Protein – A) was equal to 2,151 MoM (Multiples of Median), while β-hCG (β – chorionic gonadotropin) was equal to 1.304 MoM. Based on the above parameters and maternal age, the risk for trisomy 21 and other chromosomal abnormalities were low (1 in 8837). Therefore, no further tests were suggested.

**Fig. 2:**
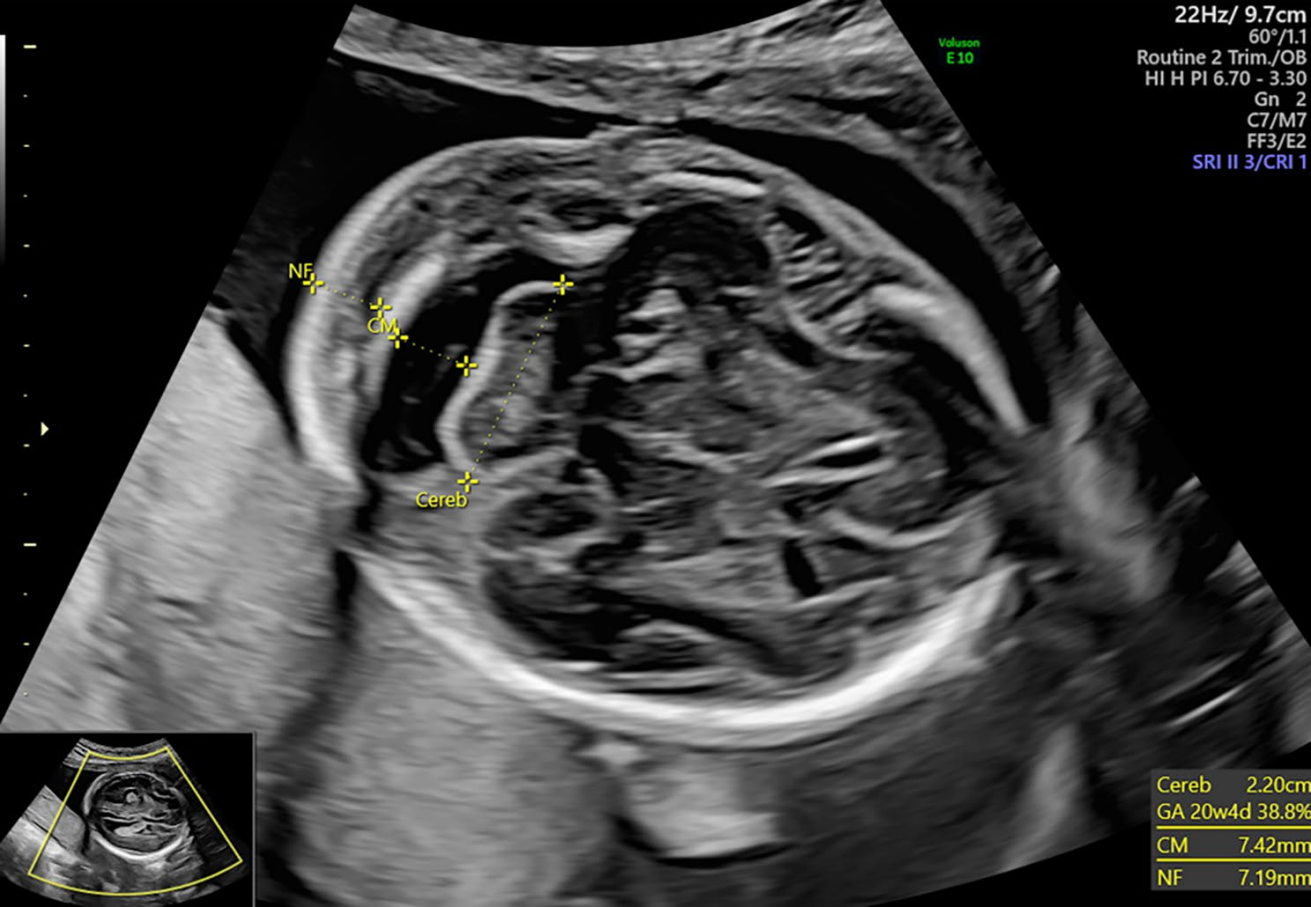
Depiction of increased NF (nuchal fold) during the Anomaly Scan (21 + 3 weeks of gestation)

**Fig. 3:**
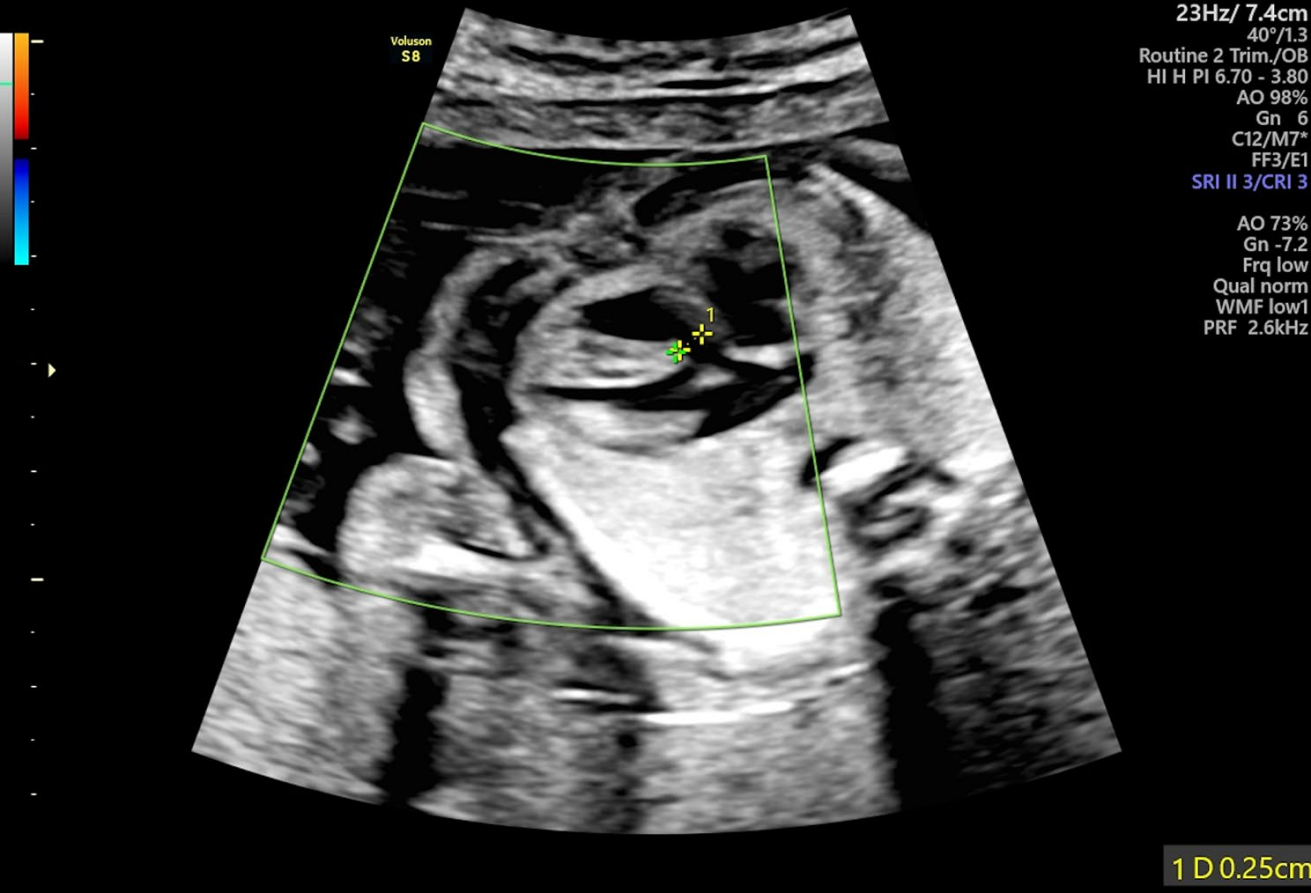
Depiction of VSD (Ventricular Septal Defect) during the Anomaly Scan (21 + 3 weeks of gestation)

**Fig. 4:**
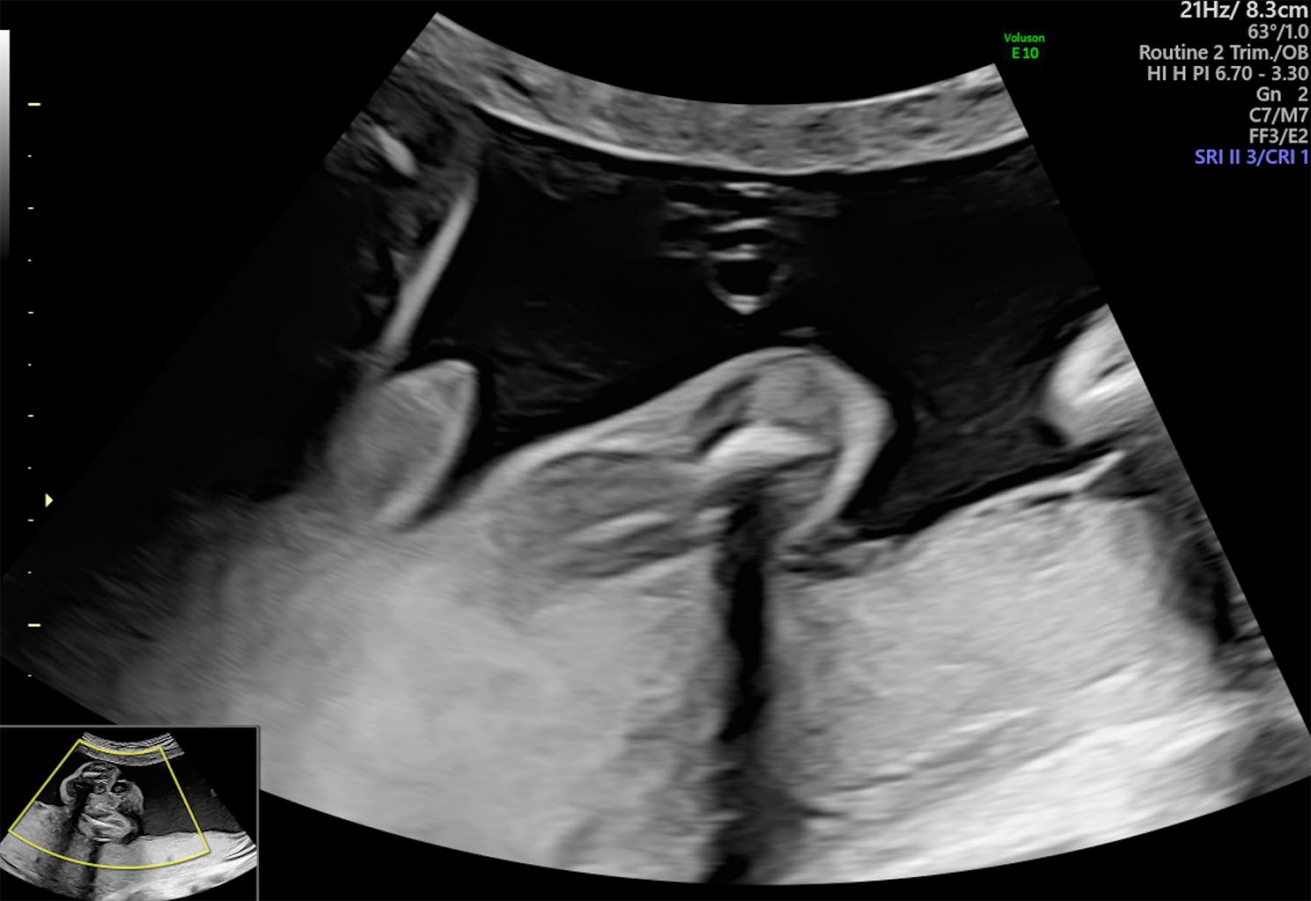
Depiction of micropenis during the Anomaly Scan (21 + 3 weeks of gestation)

During the Anomaly Scan which was performed at 21 weeks and 3 days of gestation, Estimate Fetal Weight (EFW) was on the 87th centile and fetal growth including all long bones, abdominal and head circumferences were within normal range. Regarding fetal anatomy, brain structure, spinal cord, renal and gastrointestinal systems’ anatomy was normal, and amniotic fluid volume was within normal range. However, nuchal fold was slightly increased (7,1 mm) (Fig. 2) and during assessment of cardiac anatomy a ventricular septal defect (VSD) (Fig. 3) was revealed. Finally, external male genitalia were present, complicated with micropenis (Fig. 4).

Moreover, based on the finding of VSD, parents were counseled for fetal echocardiogram, which confirmed the membranous VSD measuring 2 mm. Fetal echocardiogram also revealed restricted growth of aortic isthmus (AoI) without increased velocities (velocity on AoI: 0,76 m/sec) and normal blood flow and small pericardial perfusion without hemodynamic consequences.

Following genetic counseling from a clinical geneticist, the parents decided to terminate the pregnancy and declined autopsy.

## Results

**Fig. 5:**
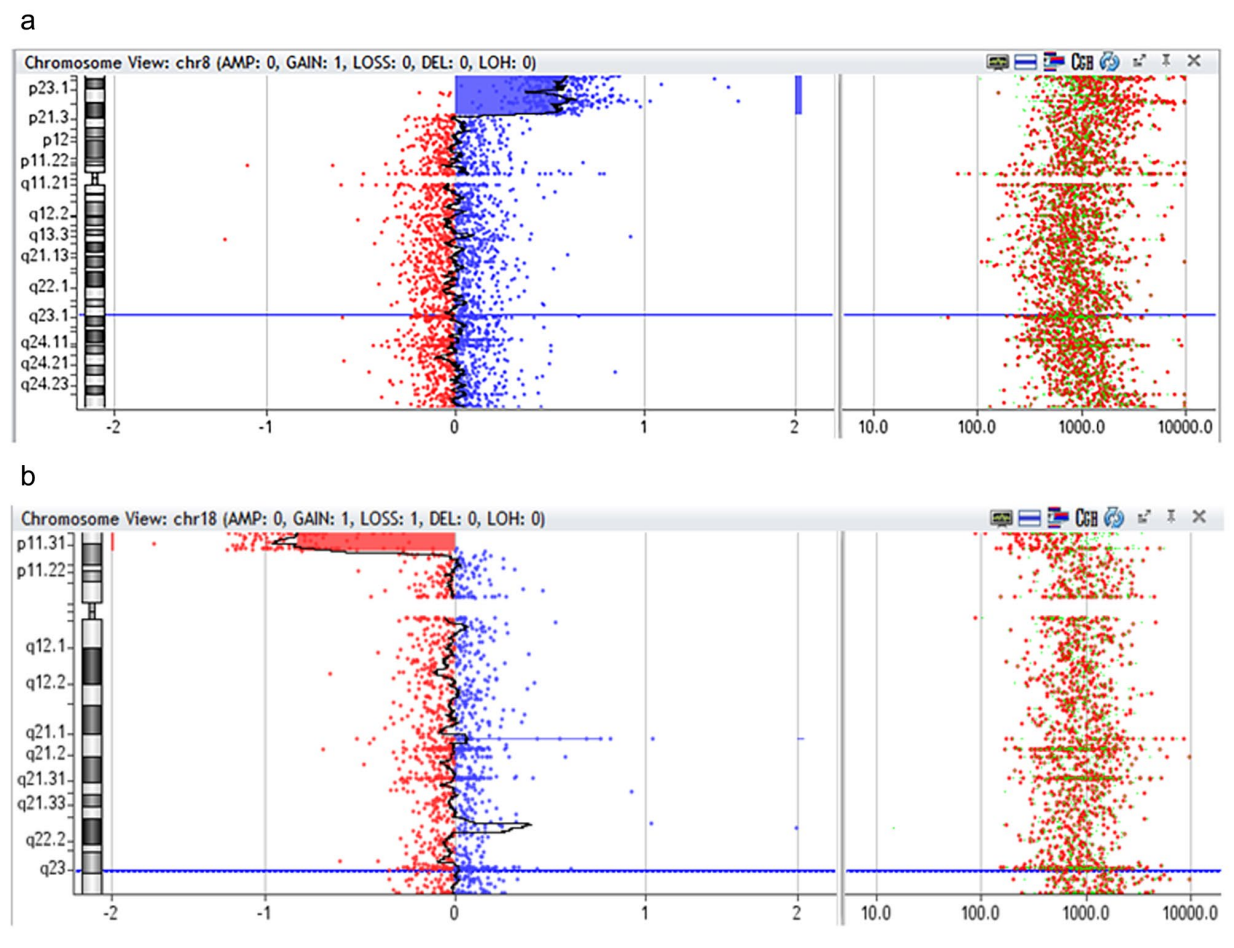
**a** array CGH of the fetus No4 showing gain of the chromosomal region 8p23.3p22 **b**: array CGH of the fetus No4, showing loss of the chromosomal region 18p11.32p11.31

**Fig. 6:**
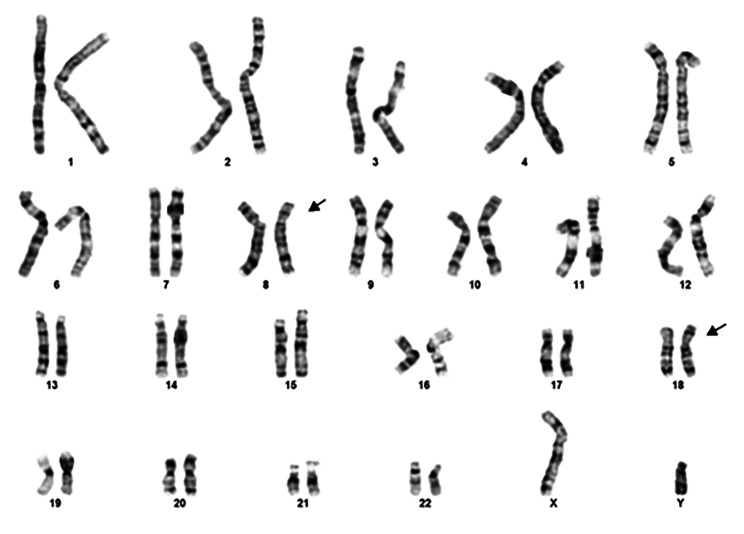
Male karyotype with a balanced reciprocal translocation between the short arm of chromosome 8 and the short arm of chromosome 18 (respective breakpoints involved 8p22 and 18p11.31, **46,XY, t(8;18)(p22;p11.31**)

Following genetic counseling the parents opted for fetal Karyotyping and an uncomplicated amniocentesis was performed. Molecular karyotyping with array-GCH (array-based comparative genomic hybridization) was performed which revealed a 17 Mb duplication in chromosomal region 8p23.3p22 and a 3,8 Mb deletion in chromosomal region 18p11.32p11.31 *(*Fig. 5a and b*).* According to the array-CGH results, the genomic profile of the fetus was determined to be: arr[hg19] 8p23.3p22(191,530_17,258,463)x3,18p11.32p11.31(148,963_3,969,941)x1. The duplicated and the deleted regions encode 56 and 17 genes registered in OMIM database, respectively. In current literature there is no report of existence of both findings in one patient. However, when assessed individually, the 8p duplication is referred as the “Trisomy 8p Syndrome” and the 18p deletion as the “Chromosome 18p Deletion Syndrome”. As both findings were met on the telomeric regions of the chromosomes and based on their obstetrical history, the parents were referred for conventional karyotyping, as the chromosomal rearrangement found in the fetus was very likely to be inherited. The fetus’s mother had a normal female karyotype (results not shown). However, conventional cytogenetic analysis of the father revealed a balanced reciprocal translocation between the short arm of chromosome 8 and the short arm of chromosome 18 (karyotype according to ISCN was 46,XY, t(8;18)(p22;p11.31) ***(***Fig. 6***).*** The finding that the chromosomal rearrangement of fetus no4 is inherited, explains also the holoprosengephaly of the fetus No1 and the spontaneous miscarriage of the second pregnancy.

## Discussion and review of the literature

### 18p deletion syndrome

The clinical features of 18p deletion syndrome vary significantly from to one patient to another [4]. It is crucial to mention that, despite identical breakpoints, the phenotype of the affected individuals remained significantly variable, indicating that many of the genes on 18p cause incomplete-penetrance phenotypes when present in hemizygous state. However, it would be an omission not to state that the most of the work which has been done focusing on the clinical and molecular definition of the syndrome, was held before the widespread use of microarray analysis, limiting the ability of a precise diagnosis [9]. Nevertheless, the genotype-phenotype correlation of the syndrome is remaining a challenge [10].

It has been reported that the 1/3 of the individuals carrying a 18p deletion, have inherited the mutation from unbalanced parental transmission of structural rearrangements (familial balance translocation, unbalanced whole-arm translocation between chromosome 18 and one of the acrocentric chromosomes -mainly 21 or 22- familial transmission or a ring chromosome) [10, 11]. In the first reports of familial transmission of the 18p deletion, there was an agreement that the inheritance could occur only due maternal structural rearrangements. However, this theory is now overcome, as it has been proved that both parents can transmit a deletion of 18p [12]. The remaining 2/3 of the cases are the result of a *de novo* terminal deletion of 18p [13]. According to Scaub et al., about 50% of 18p deletions breakpoints is located in the pericentromeric region, while the remaining cases are scattered along the short arm of chromosome 18 [14]. Additionally, it is important to be noted that as patients with 18p Deletion Syndrome are fertile, genetic counselling is crucial in order to eliminate the risk of familial transmission of the mutation [11]. In the one hand, when a fetus carries a *de novo* deletion of 18p, the risk of recurrence in the next pregnancy is low. Nevertheless, if one of the parents is a carrier, the risk of recurrence for siblings is associated with the type and the size of the structural rearrangement. If the 18p deletion is present on a homogeneous state, the risk is increased to 50%. If the parent has mosaic deletion the risk is lower [2].

The syndrome has also characteristic dysmorphic features and the most common of these are: a round face with protruding philtrum, wide mouth with short upper lip and downturned corners, short webbed neck, low posterior hairline, small mandible, large protrunding or dysplastic ears, broad and flat nasal bridge, palpebral ptosis, epicanthal folds and strabismus. Microcephaly and a high-arched palate may be also present. In addition, in the most of the cases the chest is broad with widely spaced nipples or pectus excavacum [2]. Interestingly, according to Schinzel and colleagues, the dysmorphic features might not be evident at birth, as they became more pronounce during the childhood and specifically the age of thee [15].

Growth, developmental and psychomotor retardations are also constant symptoms of the syndrome [13]. According to Wester and colleagues, patients with a deletion in the centromeric region at 18p11.1 have global developmental delay, while those who had deletion at a distal breakpoint (distal 18p11.21) present normal or marginal mental development. The degree of intellectual impairment is also in line with the length of the deleted segment [16]. Concerning the intelligence quotient (IQ) score, the patients also present a wide spectrum of scores, varying from 25 to 75. In Sebold et al. article the average IQ was 69. Moreover, the verbal performance is often more affected than the intelligence performance [9]. Finally, behavioural problems have been reported in patients with 18p Deletion Syndrome, including deficient social behavior and personality disorders [17, 18]. Difficulties with communication, self-care or social activities have been also reported (Hasi-Zogaj et al., 2015).

Brain malformations and specifically holoprosengephaly (HPE) and its microforms, is present in 10–15% of the affected individuals, involving also the forebrain and the midface [13]. HPE spectrum includes severe brain malformations such as cyclopia, cebocephaly, agenesis of corpus callosum, premaxillary agenesis and cleft lip and palate. Other milder forms or HPE microforms include hypopituitarism, minor facial dysmorphism such hypo- or hypertelorism and flat nasal bridge, absent olfactory tracts and single central incisor, without any brain abnormality [2, 19]. The responsible gene for HPE has been identified as the *TGIF*, located in 18p11.3 region. *TGIF (*Transforming growth interacting factor) is a repressor of retinoic acid regulated gene transcription. It codes for a transcription factor that competitively inhibits the binding of the retinoid X receptor (RXR) to a retinoid-responsive promotor [20]. Therefore, decreased *TGIF* levels enhance the binding of RXR and result in overactivity of retinoic acid regulated genes, simulating the effect of excessive retinoic acid exposure, which is a well-known teratogenic factor [21–23]. Moreover, a decreased activity of *TGIF* can change the Nodal/TGF-b signaling pathway, also resulting in HPE [24]. Nevertheless, the low incidence of HPE in patients with 18p deletion is seems to be due to the autosomal-dominant inheritance of the *TGIF*, its low penetrance and other environmental reasons [25]. Additionally, white matter abnormalities such as delayed myelination, sublte thinning of white matter, white matter signal abnormalities or ischemic regions, are also common among patients with 18p Deletion Syndrome [12].

Movement disorder and muscle tone abnormalities are also frequent in patients with 18p Deletion Syndrome. Dystonia is the commonest movement disorder and it can be present as focal, segmental, multifocal, or generalized dystonia, while myoclonus, chorea, tremor, tics, and ataxia are less commonly reported. The onset of dystonia varies from birth and childhood to the middle age [26]. Hypotonia is also common among patients with 18p Deletion. Moreover, a characteristic posture is evident among this population: they stand with widespread legs and the lean slightly forward [2].

Cardiac defects such as ventricular hypertrophy, ventricular septal defect, dextrocardia with situs inverus, patent ductus arteriosus, pulmonary valve stenosis, small aortic arch, atresia or stenosis of the aortic valve with or without aortic coarcation and Tetralogy of Fallot are common and they are present in about 10% of the patients carrying a 18p deletion [11, 27]. Noticeably, Vasquez et al. reported in 2003 a case with HLHS (Hypoplastic left heart Syndrome with patent dutctus arteriosus and aortic valve atresia resulting to neonatal death. The authors also stated that when 18p Deletion Syndrome is found, a detailed check for possible ischemia should be performed, as the Syndrome might cause restricted flow in abnormal coronary arteries [28].

Abnormal genitalia, such as genital hypoplasia and cryptorchidism have been also reported frequently. Hormonal abnrormalities are also met frequently; the 23% of the affected individuals have isolated GH (Growth Hormone) deficiency, while the 13% have hypopituiarism or pituitary gland hypoplasia or total pituitary gland agenesis [12, 29]. The thyroid gland is also often affected, as in patients with 18p Deletion Syndrome thyroiditis can cause either thyreotoxicosis or insufficiency of thyroid hormones. Absence or reduction of immunoglobulin A and auto-immune disorders may also be present [2].

Hasi et al. reviewed the most common findings met in individuals carrying centromeric 18p deletions and their frequency: hypotonia/mixed- tone abnormalities (84%), neonatal complications (71%), brain magnetic resonance imaging anomalies (66%), recurrent otitis media (61%), heart defects (56%), ptosis (55%), refractive errors (52%), strabismus (42%), pectus excavatum (29%), hearing loss (23%), isolated growth hormone deficiency (23%), scoliosis/kyphosis (19%), pes planus (19%), cryptorchidism (14%), panhypopituitarism or hypopituitarism (13%), seizures (13%), immunoglobulin deficiency (IgA, IgG, or IgM) (13%), holoprosencephaly or HPE microform (13%), autoimmune disorder (10%), sacral agenesis (6%), optic nerve hypoplasia (6%), congenital cataracts (6%), and myelomeningocele (3%) [12]. Concerning the life expectancy of the patients with 18p Deletion Syndrome, it is similar to the general population when they have the commonest clinical presentation of the Syndrome and in absence of the severe malformations. However, when severe brain abnormalities are present, the prognosis is poor, as the patients may die in the newborn period [2, 12].

As the prenatal diagnosis of the syndrome has low incidence and untypical intrauterine phenotype, the real challenge is the prenatal identification of the affected foetuses. Among the cases that have been diagnosed prenatally, the commonest ultrasonographic finding indicating a chromosomal abnormality was the increased nuchal translucency (INT) during the 1st trimester. Additionally, increased nuchal fold during the 2nd trimester is also an indicator of the 18p Deletion Syndrome [13, 30]. Holoprosengephaly (HPE) is also frequently depicted, while severe unilateral hydronephrosis and renal dysplasia have been also described [10, 31]. Regardless the cases with major anomalies such as HPE or INT, the prenatal diagnosis of the syndrome is accidental during NIPT (non-invasive prenatal diagnosis) or after karyotyping due to multiple congenital malformations [5] .

### 8p duplication syndrome

Chromosome 8p duplications can cause uncommon genetic disorders with varied manifestations. The vast majority of the diagnoses are set during childhood, after karyotyping due to neurodevelopmental disorders and/or intellectual disability [32]. Some people may appear normal, while others may have a variety of clinical characteristics from moderate to severe, depending on the size and area of the duplicated chromosome [33].

In the vast majority of the cases, a paternal translocation is responsible for disorder when inherited, while de novo mutations appear rarely [34]. The duplicated material from 8p region may remain on the same chromosome or, rarer can be translocated to a different one. Chromosome 8p is vulnerable to a variety of recurrent rearrangements, due to LCRs (Low copy repeats) and widespread inversion polymorphisms. According to Barber et al., the typical genomic region affected includes a 3.68 Mb region; however, a minimal region spanning 776 kb which contains eight significant genes, responsible for the different phenotype of any patient [33, 35].

Due to scientific advancements in standard cytogenetics, inverted duplications on chromosome 8p are nowadays frequently diagnosed. The cytogenetic examination should consider the possibility that chromosome 8p has a susceptibility for more complex abnormalities besides inverted duplications [36].

Nevertheless, 8p inverted duplication deletion syndrome is a distinct condition from 8p Trisomy Syndrome, which has a well-defined clinical presentation [37]. The common symptoms of 8p duplication syndrome are; craniofacial dysmorphism with high forehead, frontal or parietal bossing, mild ptosis, hypertelorism, downslanting palpebral fissures, broad nasal bridge, short prominent philtrum, carp mouth, abnormal dentition, full cheeks, and a round face. Additionally, deep palmar and plantar grooves, behavioral and psychiatric abnormalities, intellectual disability accompanied with delayed speech, language and motor development, urogenital and skeletal anomalies and also malformations of the heart and the great vessels are frequent manifestations of the syndrome. Furthermore, during the perinatal and neonatal period feeding problems, hypotonia and tracheomalakia have also been described [38].

However, the mental status among patients with 8p duplication syndrome varies considerably. Barber et al. reported four probands with 8p23.1 duplication inherited from a normal parent without any dysmorphic features or intellectual disability [32]. Furthermore, Engelen et al. had also described a case of 8p trisomy where the only finding was mild intellectual delay [37, 39]. In addition, in Shi et al. case series, the probands presented normal intellectual development and only mild delay in speech and language skills [8, 40]. Autism spectrum disorders, behavioral and phychiatric abnormalities have been also frequently reported [8, 41, 42]. Concerning brain structure malformations, agenesis of the corpus callosum and Dandy-Walker malformation have been reported [38] .

Genotype-phenotype correlation in 8p Trisomy Syndrome is also crucial for genetic counseling. The short arm of chromosome 8 contains about 484 genes [38]. Specifically, the 8p23.3p22 region contains 81 protein-coding genes. Concerning the cardiac defects, which are common among patients carrying a 8p duplication, a haploinsufficiency gene the *GATA4*, has been associated with atrial, ventricular and atrioventricular septal defect and tetralogy of Fallot.

As far as the prenatal ultrasonoographic image is concerned, congenital cardiac abnormalities are common among the affected fetuses. The 8p area is thought to be significant for laterality in embryonic heart development and has been identified as the essential region most frequently observed in cardiac malformation, notably ASD (atrial septal defect) or VSD (ventricular septal defect) [43]. Other frequently seen cardiac alterations during intrauterine life, as described in a case by Akkurt et al. are: cardiomegaly with enlarged ventricles and thickened heart walls, polyvalvular dysplasia with regutitation and dilated pulmonary arteries [6]. Abnormal cardiac axis and dextrocardia have been also reported [44, 45]. Additionally, fetuses with 8p trisomy are often growth retardated and in the vast majority of the cases, there are also abnormal Doppler studies [6]. Brain is also affected in trisomy 8p. Brain malformations which have been described are: ACC (absent corpus callosum), cerebral hypoplasia, choroid plexus cyst, enlarged 3rd ventricle and hydrocephalus [6, 44, 45] .

### Correlation of our case and other cases reported

As it has been mentioned before, this is the first case of a prenatal diagnosis of a simultaneously presence of 18p Deletion Syndrome and 8p Duplication Syndrome and the second, considering both prenatal and postnatal diagnosis. Taking as fact that both the syndromes have a very variable phenotypes which are also commonly overlap each other, it is really challenging to distinguish which of the clinical and ultrasonographic features are corresponding to 18p Deletion or 8p Trisomy Syndrome. In this article the current literature was investigated throughly for both the mutations and their main and commonest features are presented.

As the worldwide literature is concerned, Pendina et al. reported a postnatal diagnosis of the same mutations in a woman with short stature and minor facial dysmorphism, normal intelligence status, first grade obesity and several miscarriages. In that article, the authors focused in the reproductive history of the patient due to her will of carrying a healthy fetus [46] .

Jin et al. in 2021 authored an article presenting 4 cases with 18p Deletion Syndrome which all were terminated. The first fetus has holoprosengephaly, as the first pregnancy of the patient reported in this case [10]. A fetus with alobar holoprosengephaly, facial dysmprphism and 18p Deletion syndrome was reported by Chen et al. in 2011 [25]. Holoprosengephaly microforms have been reported in articles by Yank et al., and Luctosa-) [29, 47].

In 2019, Lee et al., reported a prenatal diagnosis of a 18p Deletion Syndrome which in the anomaly scan multiple abnormalities were detected, such as bilateral hydronephrosis, choroid plexus cyst, clench hand and hypertelorism. A perimembranus VSD was also detected, as in our case. This pregnancy was also terminated [31]. Also in 2019, Qi et al. presented three cases of 18p Deletion Syndrome. In the first pregnancy karyotype was performed due to increased nuchal fold as in our case. As the pregnancy continued, oligohydramnios and IUGR (intrauterine growth restriction) were developed and the pregnancy was terminated during the 34th week. The post mortem examination revealed facial dysmorphism, splenomegaly, hepatocyte hydropic degeneration, right cryptorchidism, umbilical cord edema and placental villus dysplasia. The second fetus had normal ultrasound findings throughout pregnancy, but due to increased risk for trismomy 21 arised from the biochemical results, karyotype was performed and the diagnosis of 18p Deletion Syndrome was set. The pregnancy was terminated during the 32nd week and the autopsy showed only facial dysmorphism. The third fetus had increased nuchal translucency in the first trimester ultrasound examination while in the second trimester IUGR was developed. The pregnancy was terminated during the 23rd week and the autopsy also showed only facial dysmorphism [48]. Fogu et al. and Yin et al. also reported fetuses with the 18p Deletion Syndrome which were presented increased nuchal folds or nuchal edema, as the third fetus of our case did [13, 30]. One of the initial reports of the 18p Deletion Syndromes was authored by Nakano et al. in 1977. In this case, micropenis was present, similar to the third fetus of this case, along with hypotonia, facial dysmorphism, low birth weight, fused rips, developmental and motor delay [49]. A fetus with micropenis and 18p Deletion Syndrome was also reported by Chen et al. in 2010 [25].

Concerning the articles in the current literature presenting case with 8p Trisomy Syndrome, Shi et al. and Gug et al. have reported two individual cases with ventricular septal defects (VSD) [8, 49].

As it clearly noticed, the vast majority of the ultrasonographic finding in the fetuses of the first and the third fetuses of this case are more likely to be due to the 18p mutation. Nevertheless, facial dysmorphism, cardiac defects and genital abnormalities are also frequently reported in both the syndromes.

## Conclusion

Although 18p deletion and 8p trisomy syndromes affect adversely the patient and their enviroment’s quality of life there is no specific treatment available. Therefore, the prenatal diagnosis of these syndromes is crucial. However, given the varied and untypical clinical presentation of these syndromes and also the fact that characteristic findings of the syndromes may not present during pregnancy, the prenatal diagnosis of the syndrome still presents as a challenge. Early developmental planning and intervention would optimize the developmental potential in these children. Follow-up and genetic counseling for both the children would be required at a later date. Moreover, this case highlights the importance of testing the parents when such chromosomal rearrangements are detected prenatally, since one of the parents could be a carrier of a chromosomal rearrangement. This way, appropriate genetic counseling can be offered. In similar cases, the option of prenatal diagnosis in future pregnancies should discussed with the couple, as there is a high risk of recurrence of chromosomal imbalance.

## Data Availability

For any further information or access to the data of this study, please contact Dr Anna Eleftheriades in her e-mail: anna.eleftheriadi@usb.ch.
